# Bacteriological Quality of Raw Ovine Milk from Different Sheep Farms

**DOI:** 10.3390/ani10071163

**Published:** 2020-07-09

**Authors:** Andualem Tonamo, István Komlósi, László Varga, Levente Czeglédi, Ferenc Peles

**Affiliations:** 1 Doctoral School of Animal Husbandry, University of Debrecen, 138 Böszörményi Street, 4032 Debrecen, Hungary; andytonamo@agr.unideb.hu; 2 Institute of Animal Science, Biotechnology and Nature Conservation, Faculty of Agricultural and Food Sciences and Environmental Management, University of Debrecen, 138 Böszörményi Street, 4032 Debrecen, Hungary; komlosi@agr.unideb.hu (I.K.); czegledi@agr.unideb.hu (L.C.); 3 Department of Food Science, Faculty of Agricultural and Food Sciences, Széchenyi István University, 15–17 Lucsony Street, 9200 Mosonmagyaróvár, Hungary; varga.laszlo@sze.hu; 4 Institute of Food Science, Faculty of Agricultural and Food Sciences and Environmental Management, University of Debrecen, 138 Böszörményi Street, 4032 Debrecen, Hungary

**Keywords:** sheep, ewe, ovine, milk, microbiology, bacteriology, udder surface

## Abstract

**Simple Summary:**

The aim of this study was to evaluate the bacteriological quality of sheep milk produced by four farms in Eastern Hungary. In addition to individual raw milk and bulk tank milk samples, the udder surface of ewes was also tested for bacterial counts. A total of 164 samples were collected during regular milking sessions. The results showed that bulk tank milk samples contained up to 10,000 times as many bacteria as did their individual raw milk counterparts. The mean concentrations of bacteria in bulk tank milk on two farms exceeded regulatory limits. Additional research needs to be done to clarify this.

**Abstract:**

The primary purpose of this research was to examine the bacteriological properties of raw ovine milk produced by Merino, Tsigai, Dorper, Lacaune, and British Milk Sheep flocks on four sheep farms located in the eastern part of Hungary. In addition to individual raw milk (IRM) and bulk tank milk (BTM) samples, the udder surface (US) of ewes was also tested for bacteriological quality. A total of 77 US, 77 IRM, and 10 BTM samples were collected in the early morning during regular milking sessions. The samples, kept cooled at temperatures below 4 °C, were delivered to the microbiological laboratory and were examined immediately. The relatively low numbers of bacteria in both US and IRM samples reflected good housing conditions of ewes kept on the four farms studied. However, BTM samples had up to 3.5–4.0 log_10_ CFU/mL higher mean bacterial counts than their IRM counterparts, and the mean levels of bacteria in BTM on two farms even exceeded the regulatory limit of 6.18 log_10_ CFU/mL. Further studies need to be performed to clarify this issue.

## 1. Introduction

Milking animals are not limited to cows in numerous parts of the world. With an overall annual milk production of 10.2 million metric tons, sheep are among the top four dairy animal species globally [1]. Most ovine milk is processed into cheese [2,3]. The dairy sheep industry is a promising branch of livestock production in Hungary. According to the Hungarian Central Statistical Office [4], the country had 781,700 head of ewes at the end of 2019 with the majority of the ewe population belonging to the Merino breed. In addition to Merino, some dairy breeds are also present. There are 19 dairy sheep farms in the country where milking data are recorded on a regular basis. These are Lacaune, Milking Tsigai, and British Milk Sheep farms. However, on some commercial farms, Awassi and Merino are also milked. With a quantity of 1604 metric tons, Hungary was the 15th most important sheep milk producing country in the European Union in 2018 [5]. Milk, and especially sheep milk, is not only a rich source of nutrients for humans and animals, but also an excellent medium for the growth of microorganisms due to its fat, protein, lactose, minerals, and vitamin contents [6,7].

Milk synthesized in a healthy ovine mammary gland contains relatively low numbers of microorganisms [8]. However, during and after milking, it may become colonized by a variety of microbes from the teat surface, air, feed, water, milking equipment, and other environmental sources [6]. The microbiological quality of sheep milk is largely influenced by the method of milking, breed, housing, season, stage of lactation, and farm hygiene [9]. According to Regulation (EC) no. 853/2004 of the European Parliament and the Council of the European Union [10], mesophilic bacterial count (MBC), determined at 30 °C, is an essential indicator of the microbiological quality of sheep milk. The MBC of raw ovine milk that will undergo pasteurization must not exceed 1.5 × 10^6^ CFU/mL (6.18 log_10_ CFU/mL). However, if raw sheep milk is intended for processing without heat treatment, its MBC may be, at most, 5.0 × 10^5^ CFU/mL (5.70 log_10_ CFU/mL) [10].

Significant efforts have recently been made in several countries to improve the microbiological quality of raw ovine milk. Based on data generated over a period of three years, which range from 2012 through 2014, researchers from the Czech Republic and Slovakia developed a system to greatly reduce microbial counts in sheep milk. By gradual implementation of the system, a regulatory limit of 3.0 × 10^5^ CFU/mL (5.48 log_10_ CFU/mL) for MBC in raw ewe’s milk may reasonably be set after 15 years. The corresponding threshold value in raw ovine milk intended for processing without heat treatment can be as low as 2.0 × 10^5^ CFU/mL (5.30 log_10_ CFU/mL) within the same timeframe [11].

Unpasteurized sheep milk may contain beneficial lactic acid bacteria, up to 6.0 log_10_ CFU/mL, capable of facilitating dairy fermentations and/or promoting health. In contrast, other microbes occurring in raw ewe’s milk can cause food spoilage or human diseases [6]. The consumption of raw milk from sheep may pose a risk to human health due to the potential presence of bacterial pathogens, e.g., *Brucella melitensis*, *Campylobacter* spp., *Listeria monocytogenes*, *Mycobacterium avium* subsp. *paratuberculosis*, *Salmonella* spp., *Staphylococcus aureus*, Shiga toxin-producing *Escherichia coli*, and *Yersinia pseudotuberculosis* [12,13,14]. The major bacteria of concern from a milk safety perspective include *Enterobacteriaceae* and *S. aureus* [13].

Unlike bovine milk, relatively little is known about the microbiological properties of raw milk from small ruminants in Hungary [15]. Therefore, the objective of this study was to provide data on the bacteriological quality of raw ovine milk produced by Merino, Tsigai, Dorper, Lacaune, and British Milk Sheep flocks on four sheep farms situated in the eastern part of the country. In addition to individual and bulk tank milk samples, the udder surface of animals was also tested for bacteriological quality.

## 2. Materials and Methods

### 2.1. Farms and Animals

One experimental sheep farm (F1) with three breeds and three dairy sheep farms (F2–F4) were enrolled in the study, which was carried out between March and July in 2018 and 2019. The farms were located in the eastern part of Hungary, with F1 to F3 being situated in Hajdú-Bihar County and F4 in Jász-Nagykun-Szolnok County. The flock size on each farm ranged between 51 and 292 ewes. Regular milk recording was carried out according to the Sheep Performance Testing Codex of the Hungarian Sheep and Goat Breeders’ Association [16] following the guidelines of the International Committee for Animal Recording (ICAR) [17]. Animals on F1 were kept indoors, whereas those on F2–F4 were allowed to graze during daytime. Ewes were milked in a milking parlor on both F2 and F4 and by hand on F3. In contrast, no milking was done on F1 at the time of this study. However, a milking parlor has been built since then and milking will start in the coming years. The ewes on F2–F4 were milked twice a day, i.e., early morning and late afternoon. Pre-milking disinfection was applied to teats only on F2. From F2–F4, milk was delivered to the dairy processing plant 2–3 days after milking. The main characteristics of the farms are shown in Table 1.

### 2.2. Sampling

Prior to sample collection, basic data were gathered (i.e., breed of sheep, flock size, milking time, and housing). In addition, observations were made on milking conditions, including hygienic practices and milk storage equipment used by the farms. Of the 51–292 ewes kept on each of the farms studied, 5–14 mixed-parity animals were randomly selected for individual milk and udder surface sampling (Table 2). Individual and bulk milk samples were taken on the same days. The samples were collected 2–6 weeks after weaning. However, on F1, ewes were sampled before weaning. Samples were taken from the same animals 2–3 times. All samples were collected between March and July in 2018 and 2019 from a total of 29 ewes kept on the four farms. At the very beginning of this study, environmental samples were taken (from udder and teat surfaces, barn walls, and mouth of lambs) and examined for microbiological quality. Based on the results (data not shown), only udder surface samples were collected thereafter. Forty-two udder surface (US) and 42 individual raw milk (IRM) samples from F1, 15 US, 15 IRM, and 6 bulk tank milk (BTM) samples from F2, 10 US, 10 IRM, and 3 BTM samples from F3, and 10 US, 10 IRM, and 1 BTM samples from F4 were collected and examined throughout the whole duration of the study. Thus, a total of 77 US, 77 IRM, and 10 BTM samples were tested (Table 2).

All the samples were taken in the early morning from un-milked ewes during regular milking sessions, according to the Sheep Performance Testing Codex of the Hungarian Sheep and Goat Breeders’ Association [16]. At first, US samples were collected from a 20-cm^2^ area of udders using swabs moistened with a solution of 0.1% peptone (Merck Kft., Budapest, Hungary) and 0.9% NaCl (VWR International Kft., Debrecen, Hungary). Prior to milk sampling, the teats and udders of ewes were cleaned with cotton towels soaked in 70% ethanol (Molar Chemicals, Halásztelek, Hungary). IRM samples were aseptically collected after the first four streams of milk were discarded. The samples, kept cooled at temperatures lower than 4 °C, were delivered to the microbiological laboratory at the Institute of Food Science of the University of Debrecen within 1 hour of collection and were examined immediately.

### 2.3. Bacteriological Examination

Upon arrival at the laboratory, raw milk and US samples were kept refrigerated, i.e., below 4 °C, until examination. Serial decimal dilutions were then prepared according to International Standard ISO 6887-1: 2017 [18]. The culture methods used to enumerate specific microbial groups and species are listed in Table 3.

### 2.4. Statistical Analysis

The bacterial counts, determined by the colony count technique and expressed as CFU/mL (for milk samples) or CFU/cm^2^ (for udder surface samples), were log_10_-transformed to normalize the distributions before calculating the arithmetic means and SD of these transformed counts. The effects of farm (F1–F4) and year (F1) on bacterial counts were tested by *t*-test, one-way ANOVA, and the non-parametric Kruskal–Wallis test using SPSS version 20.0 [22]. Differences were considered to be significant at *p* < 0.05. Association between major microbial quality indicators, i.e., *Enterobacteriaceae* and mesophilic bacterial counts, of US and IRM samples were evaluated by Pearson correlation.

## 3. Results

### 3.1. Bacteriological Quality of Udder Surface Samples

Table 4 shows that the mean MBC on the udder surfaces of ewes kept on F1, F2, F3, and F4 were 2.7, 2.5, 2.2, and 2.4 log_10_ CFU/cm^2^, respectively. There was a significant difference (*p* < 0.05) between F1 and F3 in this regard.

The mean *Enterobacteriaceae* counts (EBC) on the udder surfaces of individuals on F1, F2, F3, and F4 were 1.5, 1.0, 1.0, and 0.5 log_10_ CFU/cm^2^, respectively, with F1 showing significantly increased EBC (*p* < 0.05) compared to F4. As far as *S. aureus* is concerned, this species was not present at detectable levels on the udder surfaces of ewes belonging to any breed on any farm tested (Table 4).

### 3.2. Bacteriological Quality of Raw Milk Samples

As shown in Table 5, the mean MBC determined in the individual milk samples of ewes on F1, F2, F3, and F4 were 3.2, 3.3, 3.5, and 1.8 log_10_ CFU/mL, respectively. In terms of MBC, the IRM samples from F4 were superior (*p* < 0.05) to those from the other three farms, which did not differ significantly from one another (*p* > 0.05). The sheep flocks kept on F1 and F2 produced milk containing significantly different levels of *Enterobacteriaceae* (*p* < 0.05). The mean EBC of IRM samples from ewes on F3 and F4 were 2.2 and 1.4 log_10_ CFU/mL (*p* > 0.05), respectively. *S. aureus* was not found in the IRM samples collected on F1 and F2. In contrast, the raw milk of individuals kept on F3 and F4 contained *S. aureus* at 2.6 and 2.8 log_10_ CFU/mL (*p* > 0.05), respectively.

On three out of the four farms involved in this study, BTM samples were also collected and examined for bacteriological quality. As seen in Table 5 and Table 6, depending on the specific microbial group tested, BTM samples had up to 3.5–4.0 log_10_ CFU/mL higher mean bacterial counts than did their IRM counterparts.

The viable counts of mesophilic bacteria, *Enterobacteriaceae*, and *S. aureus* in US and IRM samples taken on F1 and grouped according to breed and year are indicated in Table 7. The samples collected from Tsigai ewes tended to contain the highest MBC and EBC. A similar observation was made with respect to year, in that both types of samples examined in 2018 mostly had higher (*p* < 0.05) MBC and EBC than those tested in 2019.

### 3.3. Correlations between Major Microbiological Parameters

Pearson correlation coefficients (r) were then calculated between the MBC and EBC of US and IRM samples (Table 8). The relationship between all tested pairs of bacterial quality indicators was positive. EBC^US^ showed a highly significant correlation (*p* < 0.01) with both EBC^IRM^ (r = 0.56) and MBC^US^ (r = 0.32). Unsurprisingly, EBC^IRM^ and MBC^IRM^ were also significantly correlated (r = 0.29, *p* < 0.05), and the same was true for EBC^IRM^ and MBC^US^ (r = 0.27, *p* < 0.05).

## 4. Discussion

The body of the animal, and especially the surface of her udder and teats, potentially contains a high diversity of bacteria that can gain access to milk [6]. The udder surface of ewes inevitably becomes contaminated with manure and mud when animals lie down. For this reason, US samples were collected and examined in this study. The elevated MBC in US samples from ewes on F1 compared to those on F3 must have been due to the Tsigai breed effect. This breed of sheep has a hairy udder surface, which is prone to absorb dust particles and feces. The mean MBC of US samples from F2 and F3 were similar (*p* > 0.05) because these farms did not differ in terms of hygienic and management conditions and both had Lacaune as the sole breed of sheep. Comparable data on the bacteriological quality of US samples from multiple sheep breeds are scarce in the literature, and this is the first research dealing with the microbiological status of ovine udder surfaces in the area studied.

Surprisingly low MBC were detected in the raw milk samples collected from British Milk Sheep individuals on F4, which was, at least in part, attributable to the robustness and adaptability of these strong and hardy animals. Although F2 and F3 largely differed in milking practices, i.e., parlor machine milking vs. hand milking and pre-milking teat disinfection vs. no disinfection, respectively (Table 1), the general bacteriological quality of IRM samples from Lacaune flocks was similar (*p* > 0.05) on both locations. In contrast, Bytyqi et al. [23] reported that the farm had a highly significant effect (*p* < 0.0001) on the bacterial load of raw ovine milk.

A significant difference (*p* < 0.05) was observed in EBC between the IRM samples collected from various breeds of sheep on F1 and F2. Ewes on F2 were milked in a milking parlor, and milking machines are known to contain a reservoir of microbes [24]. In addition, there was a difference in the location of F1 and F2, and this may have also affected the EBC of IRM samples. Quigley et al. [6] reported that location of the farm can influence the microbiological composition of ovine milk. The means, which ranged from 1.1 to 2.3 log_10_ CFU/mL, were over three orders of magnitude lower than those determined by Ombarak and Elbagory [25] in Egyptian raw ewe milk (i.e., 5.2 log_10_ CFU/mL). Despite the abundance of available nutrients in sheep milk, no growth of *S. aureus* was detected in any IRM sample taken on F1 and F2. Even the mean *S. aureus* count of 2.8 log_10_ CFU/mL observed on F4 was substantially lower than the corresponding mean value of 4.1 log_10_ CFU/mL reported by Ombarak and Elbagory [25].

The concentration of mesophilic bacteria in raw bulk milk reflects the level of hygienic conditions during milking and milk handling including storage. However, the MBC of raw ovine milk, even if stored at 4 °C, increases with the progress of storage time [26,27]. The mean MBC of BTM from both Lacaune flocks, on F2 and F3, failed to meet the regulatory limit of 6.18 log_10_ CFU/mL [10]. The overall bacteriological quality of raw bulk milk from F2, where ewes were milked in a milking parlor, was not superior (*p* > 0.05) to that of BTM collected on F3 from hand-milked sheep. Similarly, Zweifel et al. [27] reported that, of all the milking techniques tested, hand milking resulted in the lowest MBC in small ruminant’s bulk tank milk. Another important factor to consider in this regard is the number of animals milked on a farm because it is associated with the frequency of milk delivery to processing facilities [27]. Since there were only 51 ewes milked on F2, raw milk was typically delivered to the dairy processing plant 2–3 days after milking. In contrast, the bulk milk produced on F4 was in compliance with the criteria established by Regulation (EC) no. 853/2004 of the European Parliament and the Council of the European Union [10]. In fact, the raw milk collected from British Milk Sheep was even suitable for processing without heat treatment. Zweifel et al. [27] and Alexopoulos et al. [9] reported that ovine BTM samples collected on farms in Switzerland and Greece had a mean MBC of 6.05 log_10_ CFU/mL and 5.48 log_10_ CFU/mL, respectively. 

Elevated EBC in BTM are usually an indication of potential bacterial contamination via the udder or from the milking utensils and water supply used [8]. Increased levels of *Enterobacteriaceae*, including coliforms, in BTM may also occur when these organisms grow on milk residues left in poorly sanitized milking equipment [28].

The mean MBC of US samples from all three breeds kept on F1 were significantly higher (*p* < 0.05) in 2018 than in 2019. The observed decrease in MBC and EBC on udder surfaces in 2019 must have been due to the rising awareness of sheep farmers about the importance of high hygienic standards during milk production. The 2018 findings were shared with farm managers at the end of the year. As a result, the frequency of udder health checks was increased and early veterinary treatments were provided in 2019. The mean MBC of IRM samples collected from Merino and Tsigai ewes were also significantly different (*p* < 0.05) between years. This observation is in agreement with the finding of Gonzalo et al. [29], who reported the year to be a very significant (*p* < 0.001) source of variation for total bacterial count in ovine BTM. In our study, a highly significant correlation (r = 0.56, *p* < 0.01) was found between the EBC of US and IRM samples, which indicates that unsanitary conditions, e.g., dirty bedding, are predisposing factors for the presence of unwanted bacteria in raw sheep milk.

## 5. Conclusions

Relatively low and positively correlated bacterial counts, i.e., MBC and EBC, were found in US and IRM samples collected from ewes kept on the four farms involved in this study. However, the levels of bacteria in BTM on two farms exceeded the regulatory limit of 6.18 log_10_ CFU/mL. Further research needs to be performed to clarify this issue.

## Figures and Tables

**Table 1:** Major characteristics of the sheep farms involved in this study.

Farm	Sheep Breed Kept on Farm	Method of Milking	Grazing	Housing	Pre-Milking or Post-Milking Disinfection
F1	Merino, Tsigai, and Dorper	No milking	No	Deep litter	No
F2	Lacaune	Milking parlor	Yes	Deep litter	Pre-milking disinfection
F3	Lacaune	Hand milking	Yes	Deep litter	No
F4	British Milk Sheep	Milking parlor	Yes	Deep litter	No

**Table 2:** Number of samples collected from each farm.

Farm	Animal Sampled	Udder Surface Sample	Individual Raw Milk Sample	Bulk Tank Milk Sample
F1	14 *	42	42	0 **
F2	5	15	15	6
F3	5	10	10	3
F4	5	10	10	1
Total	29	77	77	10

* 8 animals (4 Tsigai, 2 Dorper, and 2 Merino ewes) in 2018 and 6 animals (2 Tsigai, 2 Dorper, and 2 Merino ewes) in 2019. ** On F1, ewes were not commercially milked during the study and, therefore, only individual raw milk samples were taken.

**Table 3:** Determination of microbial counts in raw milk and udder surface samples.

Microorganism Enumerated	Method	Reference
Mesophilic microorganisms (bacteria)	ISO 4833-1:2013	[19]
*Enterobacteriaceae*	ISO 21528-2:2017	[20]
*Staphylococcus aureus*	EN ISO 6888-1:1999	[21]

**Table 4:** Bacteriological quality of udder surface samples according to the farm.

Farm	Bacteriological Parameter Tested *		
	MBC	EBC	SAC
F1 (*n* = 42)	2.7 ± 1.0 ^a^	1.5 ± 0.8 ^a^	ND
F2 (*n* = 15)	2.5 ± 0.7 ^ab^	1.0 ± 0.5 ^ab^	ND
F3 (*n* = 10)	2.2 ± 0.5 ^b^	1.0 ± 0.1 ^ab^	ND
F4 (*n* = 10)	2.4 ± 0.4 ^ab^	0.5 ± 0.3 ^b^	ND

* Values are log_10_ CFU/cm^2^ means ± SD. ^a,b^ Means within a column with different lowercase superscripts differ (*p* < 0.05). MBC: mesophilic bacteria count. EBC: *Enterobacteriaceae* count. SAC: *Staphylococcus aureus* count. ND: not detected.

**Table 5:** Bacteriological quality of individual raw milk samples according to the farm.

Farm	Bacteriological Parameter Tested *		
	MBC	EBC	SAC
F1 (*n* = 42)	3.2 ± 1.1 ^a^	1.1 ± 0.3 ^b^	ND
F2 (*n* = 15)	3.3 ± 1.0 ^a^	2.3 ± 0.8 ^a^	ND
F3 (*n* = 10)	3.5 ± 0.9 ^a^	2.2 ± 0.0 ^ab^	2.6 ± 0.7 ^a^
F4 (*n* = 10)	1.8 ± 0.4 ^b^	1.4 ± 0.0 ^ab^	2.8 ± 0.3 ^a^

* Values are log_10_ CFU/mL means ± SD. ^a,b^ Means within a column with different lowercase superscripts differ (*p* < 0.05). MBC: mesophilic bacteria count. EBC: *Enterobacteriaceae* count. SAC: *Staphylococcus aureus* count. ND: not detected.

**Table 6:** Bacteriological quality of bulk tank milk samples according to the farm.

Farm	Breed of Sheep	Bacteriological Parameter Tested *		
		MBC	EBC	SAC
F2 (*n* = 6)	Lacaune	7.4 ± 0.6 ^a^	5.1 ± 0.9 ^a^	ND
F3 (*n* = 3)	Lacaune	6.3 ± 0.4 ^a^	5.6 ± 0.9 ^a^	3.4 ± 0.6
F4 (*n* = 1)	British Milk Sheep	5.2	3.9	ND

* Values are log_10_ CFU/mL means ± SD. ^a^ Means within a column with identical lowercase superscripts do not differ (*p* > 0.05). MBC: mesophilic bacterial count. EBC: *Enterobacteriaceae* count. SAC: *Staphylococcus aureus* count. ND: not detected.

**Table 7:** Bacteriological quality of udder surface and individual raw milk samples on farm F1 according to breed of sheep and year of examination.

Origin of Sample	Parameter Tested	Merino		Tsigai		Dorper	
		2018 (*n* = 6)	2019 (*n* = 6)	2018 (*n* = 12)	2019 (*n* = 6)	2018 (*n* = 6)	2019 (*n* = 6)
Udder surface (log_10_ CFU/cm^2^) *	MBC	3.8 ± 0.5 ^a^	1.9 ± 0.4 ^b^	4.0 ± 0.7 ^a^	2.1 ± 0.5 ^b^	3.5 ± 0.7 ^a^	2.0 ± 0.3 ^b^
	EBC	1.1 ± 0.2 ^a^	0.9 ± 0.3 ^a^	2.2 ± 0.9 ^a^	0.2 ± 0.1 ^b^	1.7 ± 0.2	ND
	SAC	ND	ND	ND	ND	ND	ND
Individual raw milk (log_10_ CFU/mL) *	MBC	3.6 ± 1.3 ^a^	2.3 ± 0.8 ^b^	3.8 ± 1.1 ^a^	3.2 ± 1.2 ^b^	3.3 ± 0.9 ^a^	3.3 ± 0.5 ^a^
	EBC	ND	1.0 ± 0.3	ND	ND	ND	1.4 ± 0.0
	SAC	ND	ND	ND	ND	ND	ND

* Values are means ± SD. ^a,b^ Sub-column means within a row and breed with different lowercase superscripts differ (*p* < 0.05). MBC: mesophilic bacteria count. EBC: *Enterobacteriaceae* count. SAC: *Staphylococcus aureus* count. ND: not detected.

**Table 8:** Matrix of Pearson correlation coefficients (r) between major microbial quality indicators of udder surface samples (*n* = 77) and individual raw milk samples (*n* = 77).

Parameter	MBC^IRM^	MBC^US^	EBC^IRM^	EBC^US^
MBC^IRM^	1	0.18	0.29 *	0.02
MBC^US^	0.18	1	0.27 *	0.32 **
EBC^IRM^	0.29 *	0.27 *	1	0.56 **
EBC^US^	0.02	0.32 **	0.56 **	1

* *p* < 0.05. ** *p* < 0.01. MBC: mesophilic bacterial count. EBC: *Enterobacteriaceae* count. ^IRM^: individual raw milk. ^US^: udder surface.
